# Risk factors for inadvertent intraoperative hypothermia in patients undergoing laparoscopic surgery: A prospective cohort study

**DOI:** 10.1371/journal.pone.0257816

**Published:** 2021-09-23

**Authors:** Huai-Ying Chen, Li-Jing Su, Hang-Zhou Wu, Hong Zou, Rong Yang, Yi-Xia Zhu

**Affiliations:** 1 Department of Nursing, Ningde Municipal Hospital of Ningde Normal University, Ningde, China; 2 The School of Nursing, Fujian Medical University, Fuzhou, China; 3 Department of Medical Insurance, Fujian Medical University Union Hospital, Fuzhou, China; 4 Department of Clinical Laboratory, Fujian Medical University Union Hospital, Fuzhou, China; 5 Department of Medical Record Management, Fujian Medical University Union Hospital, Fuzhou, China; 6 Department of Anesthesiology, Ningde Municipal Hospital of Ningde Normal University, Ningde, China; Ohio State University Wexner Medical Center Department of Surgery, UNITED STATES

## Abstract

**Background:**

Inadvertent intraoperative hypothermia is frequent during open surgeries; however, few studies on hypothermia during laparoscopic abdominal surgery have been reported. We aimed to investigate the incidence and risk factors for hypothermia in patients undergoing laparoscopic abdominal surgery.

**Methods:**

This single-center prospective cohort observational study involved patients undergoing laparoscopic surgery between October 2018 and June 2019. Data on core body temperature and potential variables were collected. A multivariate logistic regression analysis was performed to identify the risk factors associated with hypothermia. A Cox regression analysis was used to verify the sensitivity of the results.

**Results:**

In total, 690 patients were included in the analysis, of whom 200 (29.0%, 95% CI: 26%−32%) had a core temperature < 36°C. The core temperature decreased over time, and the incident hypothermia increased gradually. In the multivariate logistic regression analysis, age (OR = 1.017, 95% CI: 1.000–1.034, *P* = 0.050), BMI (OR = 0.938, 95% CI: 0.880–1.000; *P* = 0.049), baseline body temperature (OR = 0.025, 95% CI: 0.010–0.060; *P <* 0.001), volume of irrigation fluids (OR = 1.001, 95% CI: 1.000–1.001, *P* = 0.001), volume of urine (OR = 1.001, 95% CI: 1.000–1.003, *P* = 0.070), and duration of surgery (OR = 1.010, 95% CI: 1.006–1.015, *P* < 0.001) were significantly associated with hypothermia. In the Cox analysis, variables in the final model were age, BMI, baseline body temperature, volume of irrigation fluids, blood loss, and duration of surgery.

**Conclusions:**

Inadvertent intraoperative hypothermia is evident in patients undergoing laparoscopic surgeries. Age, BMI, baseline body temperature, volume of irrigation fluids, and duration of surgery are significantly associated with intraoperative hypothermia.

## Introduction

Hypothermia, defined as a core body temperature < 36°C (96.8°F) [1], has an incidence of 37.5–77.2% during the perioperative period [2–4]. Body temperature decreases by 1–3°C after anesthesia induction and could reach an equilibrium state after 3–4 h [5]. Patients who experience even a mild degree of hypothermia have adverse complications, such as surgical site infections, cardiovascular adverse events [6], decreased blood coagulation function [7], prolonged anesthesia recovery time [8], and increased mortality [9].

Both environmental and medical factors are associated with the development of perioperative hypothermia [10]. Medical literature reporting on hypothermia have mostly focused on open surgeries [11–14]; however, the risk factors found in the few available reports have been inconsistent [13,15]. Compared with an open surgery, a video-assisted endoscopic surgery requires a smaller incision; nonetheless, hypothermia has been noted in patients undergoing endoscopic surgeries [16]. Video-assisted endoscopic surgeries are performed with different surgical approaches, such as the use of carbon dioxide (CO_2_) insufflation and irrigation. Since the surgical approaches vary between open and video-assisted surgeries, the risk factors for hypothermia may differ. Interventions that could reduce the risk of hypothermia are necessary but usually labor-intensive and costly. To provide targeted interventions and promote the postoperative rehabilitation of patients, a better understanding of the factors associated with hypothermia in endoscopic surgeries is warranted.

Studies on the risk factors for hypothermia in arthroscopic and thoracoscopic surgeries have been reported [17,18]. To the best of our knowledge, no study has examined the risk factors for hypothermia in laparoscopic abdominal surgeries. Therefore, our study aimed to investigate the incidence and risk factors for hypothermia in a Chinese population undergoing laparoscopic abdominal surgeries.

## Materials and methods

### Ethical approval

This study was approved by the Institutional Review Board of the Ningde Normal University Ningde Hospital (approval number 20181104) and registered at the Chinese Clinical Trial Registry (ChiCTR2100042766). The requirement for informed consent was waived by the institutional review board. The research process in the study strictly complied with the regulations of the ethics committee. All individual information was anonymized before the data analyst accessed them. No individual information was disclosed in this research.

### Study population and sampling

This was a single-center prospective cohort observational study. Patients who underwent laparoscopic abdominal surgery between October 2018 and June 2019 were enrolled. The sample size in this study was calculated using the PASS (Power Analysis and Sample Software). As our previous investigation showed a hypothermia incidence probability of 24%, we would be able to estimate this rate to be ± 4% with 95% confidence interval (two-sided). This result in around 440 patients.

The inclusion criteria were as follows: 1) age ≥ 18 years; 2) preoperative body temperature ≥ 36°C; and 3) planning for elective surgery. The exclusion criteria were as follows: 1) laparoscopic surgery converted to open surgery and 2) history of hyperthyroidism or other metabolic diseases.

### Anesthetic and operating procedure information

The surgery was performed in operating rooms with laminar airflow, and patients were provided with surgical draping and unheated cotton blankets. The ambient temperature was maintained at 22–24°C while the humidity was maintained at 50–60%. All patients included in this study received general anesthesia during the surgery. The anesthesia was started by intravenous induction with propofol, sufentanil or remifentanil and rocuronium or atracurium. Tracheal intubation was performed to make patients breathe independently throughout anesthesia. Anesthesia was maintained with total intravenous anesthesia using propofol and remifentanil or sufentanil or sevoflurane anesthesia with supplemental remifentanil and propofol infusion. Dexmedetomidine and alfentanil were used during surgery, as was the custom of the anesthesiologist. Active warming was not performed unless the core body temperature had dropped to 36°C (96.8°F). Warming the irrigation fluid to 40°C was the active approach adopted during the surgery.

### Collected variables

Intraoperative hypothermia was defined as a core body temperature < 36°C (96.8°F) detected at any time throughout the surgery [1]. Immediately upon entering the operating room, the patient’s baseline core temperature was measured from the tympanic membrane using an infrared ear thermometer (ThermoScan 5-IRT6020; Braun, Kronberg, Germany). The intraoperative temperature was monitored continually and recorded at 30-min intervals from anesthesia induction to the end of the surgery, using nasopharyngeal temperature probes (Temperature Probe 400 series; General Electric, Helsinki, Finland).

Based on a review of literatures, this study focused on the risk factors associated with preoperative variables (age, body mass index [BMI], baseline body temperature, systolic blood pressure [SBP], heart rate before anesthesia induction, and American Society of Anesthesiologists [ASA] physical status) and perioperative variables (volume of carbon dioxide [CO_2_], volume of irrigation fluid, total volume of intravenous fluid, volume of urine, blood loss during surgery, duration of anesthesia, and duration of surgery). The duration of anesthesia was defined as the time interval between the anesthesia induction and extubation of endotracheal tubes. The duration of surgery was defined as the time interval between anesthesia induction and the moment patient left the operating room. Data regarding the anesthetics were collected from the anesthesia documentation, and surgery data were collected from the electronic database on the server. Demographic, diagnosis, and previous medical data were collected from the electronic medical record system before the surgery.

### Statistical analysis

The incidence of hypothermia in the study population was estimated. Categorical variables such as department, sex, active warming, and ASA physical status were presented as numbers and percentages; continuous variables such as age, BMI, duration of anesthesia, and duration of surgery were presented as means and standard deviation (SD). Demographic characteristics between the hypothermia and normothermia groups were compared using the Chi-square test for categorical variables and the unpaired *t*-test for continuous variables. To identify the risk factors for hypothermia, a multivariate logistic regression analysis with backward stepwise selection process including all the potential variables was performed. The time of the first episode of hypothermia was recorded. Since development of hypothermia was a time depending process, a Cox regression analysis with backward stepwise selection process was used to verify the sensitivity of the results. Active warming and blood transfusion were used as adjustment variables. The results of odd ratio (OR) values by logistic regression and hazard ratio (HR) values by Cox regression and 95% confidence intervals (95% CI) were presented. A result was considered statistically significant as *P* ≤ 0.05. All analyses were performed using IBM SPSS 22.0 (IBM Corp; Armonk, NY).

## Results

### General characteristics

**Fig 1:**
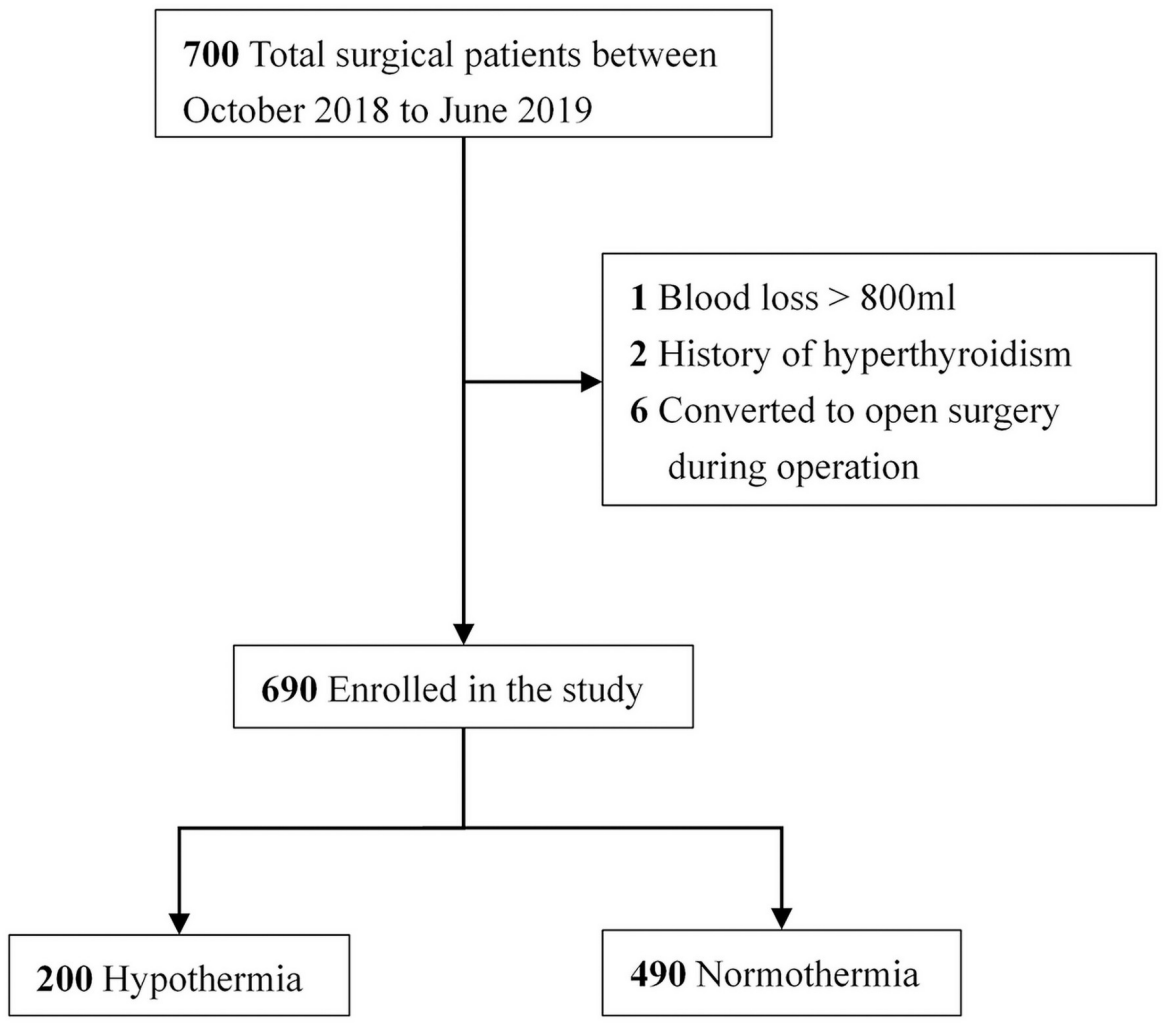
Patients enrolled in the study. A total of 690 patients, 200 with hypothermia and 490 with normothermia, were included in the final analysis.

A total of 700 patients who underwent laparoscopic surgery were included in this study. Four patients with a history of hyperthyroidism and six patients converted from laparoscopic to open procedure were excluded. Finally, 690 patients were included in the analysis (Fig 1).

Overall, 200 of the 690 (29%, 95% CI: 26%−32%) patients experienced hypothermia. Only nine patients received active warming during surgery. Majority of the patients were female (72.6%). The overall mean (SD) age of the population was 45.6 (13.33) years, and the mean (SD) duration of surgery was 139.3 (66.37) min (Table 1). Compared to those with normothermia, patients with hypothermia were older (*P* < 0.001); received more CO_2_ (*P* < 0.001), irrigation fluid (*P* < 0.001), and intravenous fluid (*P* < 0.001); and experienced longer durations of anesthesia (*P* < 0.001) and surgery (*P* < 0.001) (Table 1).

**Table 1:** Patient characteristics of the study.

Characteristic	Overall (n = 690)	Hypothermia (n = 200)	Normothermia (n = 490)	*P* value
Department				0.209 ^a^
General Surgery	274 (39.7)	70 (35.0)	204 (41.6)	
Gynecology	386 (55.9)	118 (59.0)	268 (54.7)	
Urology	20 (2.9)	7 (3.5)	13 (2.7)	
Oncology	10 (1.4)	5 (2.5)	5 (1.0)	
Sex				0.174 ^a^
Male	189 (27.4)	62 (31.0)	127 (25.9)	
Female	501 (72.6)	138 (69.0)	363 (74.1)	
Age, mean (SD), year	45.6 (13.33)	49.1 (14.05)	44.2 (12.77)	<0.001^c^
BMI, mean (SD), (kg/m^2^)	23.0 (3.25)	22.8 (3.05)	23.1 (3.32)	0.395 ^c^
Active warming				0.291^b^
Yes	9 (1.3)	4 (2.0)	5 (1.0)	
No	681 (98.7)	196 (98.0)	484 (99.0)	
ASA classification				0.671^b^
I	156 (22.6)	41 (20.5)	115 (23.5)	
II	529 (76.7)	158 (79.0)	371 (75.7)	
III	5 (0.7)	1 (0.5)	4 (0.8)	
SBP before surgery, mean (SD), (mmHg)	131.5 (18.77)	133.5 (20.52)	130.7 (17.95)	0.070 ^c^
Heart rate before surgery, mean (SD), (beat/min)	77.8 (12.27)	75.3 (11.12)	78.9 (12.58)	0.001 ^c^
Volume of CO_2_, mean (SD),(L)	203.6 (234.44)	308.5 (339.72)	160.6 (155.06)	<0.001 ^c^
Volume of irrigation, mean (SD),(mL)	584.8 (691.52)	796.0 (821.15)	498.4 (611.03)	<0.001 ^c^
Total volume of intravenous fluid, mean (SD), (mL)	1037.2 (374.42)	1197.9 (482.53)	971.4 (296.19)	<0.001 ^c^
Volume of urine,mean (SD),(mL)	160.4 (167.84)	224.8 (202.77)	134.0 (143.35)	<0.001 ^c^
Blood loss, mean (SD),(mL)	58.5 (182.71)	77.9 (249.49)	50.6 (146.47)	0.084 ^c^
Duration of anesthesia, mean (SD), minute	117.5 (63.78)	152.5 (80.86)	103.2 (48.63)	<0.001 ^c^
Duration of surgery, mean (SD), minute	139.3 (66.37)	173.9 (81.97)	125.2 (52.75)	<0.001 ^c^

Abbreviation: BMI, body mass index; ASA, American Society of Anesthesiologists physical status; SBP, systolic blood pressure; CO_2_, carbon dioxide; SD, standard deviation.

^a^ Chi-square test.

^b^ Fisher’s test.

^c^ unpaired *t*-test.

**Fig 2:**
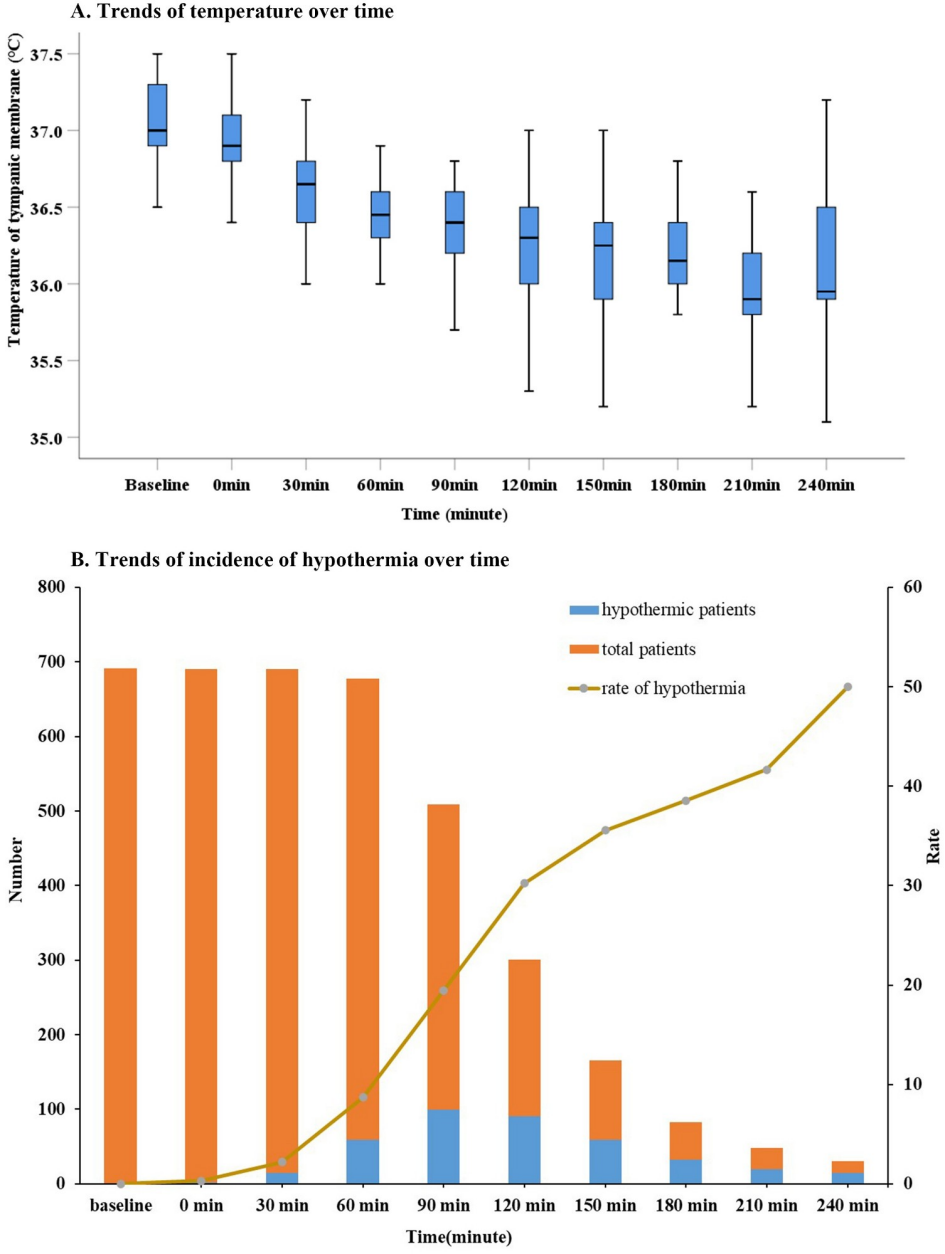
Trends of the core temperature and the incidence of hypothermia. (A)Trends of the body core temperature. (B) Trends of the incidence of hypothermia. The temperatures were monitored continually and recorded at 30-minute intervals after anesthesia induction of until the end of the surgery.

Fig 2A displays the core temperature changes over time; a decreasing tendency was observed. At the 60-min time point, the mean temperature was 36.6°C, having dropped by 0.6°C from the onset of the surgery. At the 240-min time point, the mean temperature was 35.1°C, having dropped by 1°C from the onset of the surgery. Fig 2B demonstrates the gradual increase in the number and incidence of hypothermia over time. At the 60-min time point, 59 patients developed hypothermia with an incidence rate of 8.7% (59/678). At the 240-min time point, 15 patients developed hypothermia with an incidence rate of 50% (15/30).

### Factors associated with hypothermia

Since the duration of surgery included the duration of anesthesia and there was a high correlation between those two factors (Pearson’s correlation coefficient: 0.962, *P* < 0.001), we retained the variable of duration of surgery in the multivariable analysis. Multivariate logistic regression analysis showed that age (OR = 1.017, 95% CI: 1.000–1.034, *P* = 0.050), volume of irrigation fluids (OR = 1.001, 95% CI: 1.000–1.001, *P* = 0.001), volume of urine (OR = 1.001, 95% CI: 1.000–1.003, *P* = 0.070), and duration of surgery (OR = 1.010, 95% CI: 1.006–1.015, *P* < 0.001) increased the risk for hypothermia. In contrast, higher BMI (OR = 0.938, 95% CI: 0.880–1.000; *P* = 0.049) and baseline body temperature (OR = 0.025, 95% CI: 0.010–0.060; *P*<0.001) prevented patients from developing hypothermia during the laparoscopic surgery. No significant relationships were observed between hypothermia and SBP, heart rate before anesthesia induction, ASA physical status, volume of CO2, total volume of intravenous fluid, and volume of blood loss (Table 2).

**Table 2:** Risk factors for inadvertent hypothermia on multivariate logistic regression analysis.

Variable	OR	95% CI	*P* value
Age (year)	1.017	1.000–1.034	0.050
BMI (kg/m^2^)	0.938	0.880–1.000	0.049
Baseline body temperature	0.025	0.010–0.060	<0.001
Volume of irrigation (mL)	1.001	1.000–1.001	0.001
Volume of urine (mL)	1.001	1.000–1.003	0.070
Duration of surgery (min)	1.010	1.006–1.015	<0.001

Variables into multivariable logistic analysis included: Age, BMI, SBP, baseline body temperature, heart rate before anesthesia induction, ASA physical status, volume of irrigation, volume of CO_2_, total volume of intravenous fluid, volume of urine, volume of blood loss, duration of surgery.

Adjusted for active warming and blood transfusion.

Abbreviation: BMI, body mass index; SBP, systolic blood pressure, ASA, American Society of Anesthesiologists physical status, CO_2_, carbon dioxide, OR, odd ratio.

Cox regression analysis showed that age (HR = 1.014, 95% CI: 1.002–1.027; *P* = 0.026), BMI (HR = 0.930, 95% CI: 0.888–0.974; *P* = 0.002), baseline body temperature (HR = 0.185, 95% CI: 0.121–0.284; *P* < 0.001), volume of irrigation (HR = 1.000, 95% CI: 1.000–1.000; *P* = 0.055), blood loss (HR = 1.001, 95% CI: 1.000–1.001; *P* = 0.033), and duration of surgery (HR = 0.991, 95% CI: 0.989–0.994; *P <* 0.001) were retained in the model. SBP, heart rate before anesthesia induction, ASA physical status, volume of CO2, total volume of intravenous fluid, and volume of urine were not included (Table 3).

**Table 3:** Risk factors for inadvertent hypothermia on multivariable Cox regression analysis.

Variable	HR	95% CI	*P* value
Age (year)	1.014	1.002–1.027	0.026
BMI (kg/m^2^)	0.930	0.888–0.974	0.002
Baseline body temperature	0.185	0.121–0.284	<0.001
Volume of irrigation (mL)	1.000	1.000–1.000	0.055
Blood loss (mL)	1.001	1.000–1.001	0.033
Duration of surgery (min)	0.991	0.988–0.994	<0.001

Variables into multivariable Cox analysis included: Age, BMI, SBP, heart rate before anesthesia induction, ASA physical status, baseline body temperature, volume of irrigation, volume of CO_2_, total volume of intravenous fluid, volume of urine, volume of blood loss, duration of surgery.

Adjusted for active warming and blood transfusion.

Abbreviation: BMI, body mass index; SBP, systolic blood pressure, ASA, American Society of Anesthesiologists physical status, CO_2_, carbon dioxide; HR, hazard ratio.

## Discussion

In our study, the incidence of hypothermia was 29.0% in the whole population. A gradual decrease in the core temperature was observed after anesthesia induction. The intraoperative core body temperature dropped by approximately 0.6°C and 1°C at the 60-min and 240-min time points, respectively. At the 60-min time point during the surgery, the incidence of hypothermia was 8.7%, and increased to 50% by the 240-min time point. This suggests that core body temperature should be closely monitored, and protective measures should be taken after anesthesia induction, especially in patients undergoing prolonged surgical procedures.

This study revealed that the factors associated with hypothermia in patients undergoing laparoscopic abdominal surgery included age, BMI, baseline body temperature, the volume of irrigation, and the duration of the surgery. Body temperature was change overtime, and hypothermia was a time-to-event outcome. A multivariate logistic regression dose not adjust for time, it may yield different estimations of the associations. Therefore, we used a Cox regression model to analyze covariate information that changed overtime. However, the results of the logistic regression and Cox regression were similar. The only difference was that volume of urine in the logistic regression model was replaced by the blood loss in the Cox regression analysis.

In the logistic regression analysis of the present study, age was a risk factor for hypothermia, which agreed with findings from other studies on video-assisted thoracoscopic surgeries [18]. The decline in metabolism in older individuals contributes to a decrease in the capacity for temperature regulation, increasing the susceptibility to hypothermia [19]. It has been reported that the risk for hypothermia increases by 1.61 times in patients ≥ 65 years [9]. Since evidence showed that mild hypothermia during surgery increases the possibility of blood loss and transfusion requirements [20], strengthen core temperature monitoring and initiate early active warming in older individuals are needed.

In the present study, higher BMI prevented patients from developing hypothermia, in accordance with previous research findings [21]. Based on the gradient theory, metabolic heat flows from the core to the periphery, and then, to the environment; therefore high BMI may have a protective effect, as the adipose tissues buffer heat transfer during the procedure [3,22]. However, this result should be interpreted cautiously as excessive weight is associated with a higher frequency of cardiovascular diseases [23].

In open surgery, the body tends to lose heat through evaporation [24]. A smaller incision was needed in laparoscopic surgery than that in open surgery, thus effectively preventing evaporative heat dissipation. It has been reported that intraoperative heat loss is mainly through radiation and convection [10]. However, in our study, the volume of CO_2_ was not associated with hypothermia; which was inconsistent with the finding from a previous study [25]. Convection is a method of heat transfer through mass motion, which may explain the fact that increased volumes of CO_2_, irrigation fluid, intravenous fluid, and urine contributed to the risk of hypothermia in this study. A large amount of CO_2_ is insufflated into the body during laparoscopic surgery, especially in cases of abdominal surgery. A meta-analysis revealed that in laparoscopic abdominal surgery, the core temperature was significantly lower in the cold CO_2_ groups than that in the heated, humidified CO_2_ groups [26]. An experiment on mice showed that humidified-warm CO_2_ preserved normothermia, and facilitated tissue repair [27]. In our study, CO_2_ insufflation did not increase the risk for hypothermia, although it was not warmed. However, we believe that humidified-warm CO_2_ should be used, as the mean temperature decreased to 35.1°C, and the incidence of hypothermia increased to 50% at the 240-min time point.

A substantial proportion of cold-fluid infusion and discharge from the body remove massive heat. In a patient weighing 70 kg, the mean body temperature may reduce by 0.25°C for each liter of fluid infused at an ambient temperature [10]. The core body temperature was approximately 1°C lower in the group that received irrigation fluids at an ambient temperature than that in the group that received irrigation fluids warmed to 39°C [28]. In the present study, irrigation and intravenous fluids were used routinely at ambient temperature unless an episode of hypothermia. Compared with intravenous fluid, the volume of the irrigation fluids was more significantly associated with hypothermia. However, results from a meta-analysis revealed that the use of warmed intravenous fluids effectively maintained the core temperature at 0.5°C higher than when intravenous fluids at an ambient room temperature were used. However, no significant differences were observed between the used of irrigation fluids at warmed and those at ambient room temperatures [29]. In our study, warming the irrigation fluid to 40°C after hypothermia had little effect on raising the core body temperature back to normal.

In this study, a longer duration of surgery increased the risk for hypothermia. This observation was consistent with results from most studies on surgical populations [18,30,31]. Heat loss begins from surgical skin preparation [32], and continues in the first hour after induction of anesthesia, as the core temperature usually decreases by 1–1.5°C [33]. A longer duration of surgery increases the time the patient is exposed to the ambient temperature and receives more unwarmed CO_2_, irrigation fluids, and intravenous fluids, which contribute to the fall in the core temperature. However, once the core temperature was low to 34.5°C, it would no longer decrease, regardless of the duration of the surgery [10].

Although we observed that factors such as age and longer duration of surgery were associated with intraoperative hypothermia, it should be noted that fewer interventions can be implemented with these factors. Since higher baseline core temperature effectively protected patients against intraoperative hypothermia in our study, and the same effect was obtained in previous studies [13,31], measures can be taken to increase the patient’s baseline core body temperature to an appropriate range before anesthesia. Recently, active warming approaches, such as the use of forced air, have focused mostly on warming the skin surface [34]. These approaches are attractive as the skin can be warmed safely, and most studies tend to support the effectiveness of these approaches [34–37]. Since most forced air devices are costly, more affordable measures of maintaining perioperative normothermia are worth exploring.

This study was a snapshot of a tertiary hospital in China. To the best of our knowledge, this is the first report on inadvertent intraoperative hypothermia developing from laparoscopic abdominal surgery in a Chinese population. However, several limitations of our study should be noted. First, the incidence of hypothermia might have been underestimated due to the 30-min period of core temperature acquisition. Shortening the intervals to 15-min might lead to a higher incidence. Second, the anesthetics used in this study varied according to the anesthesiologists’ custom, and the doses between them could not be accurately converted. Standardizing anesthetic techniques would be beneficial in capturing more variables for analysis. Third, being an observational study, confounding factors and biases might have been obscured. Finally, this was a single-center study; therefore, generalization of our findings to other populations should be done with caution.

## Conclusions

Inadvertent intraoperative hypothermia was common in patients who underwent laparoscopic abdominal surgery. Throughout the surgery, the core temperature tended to decrease, and the incidence of hypothermia tended to increase over time. Age, BMI, baseline body temperature, volume of irrigation fluids, and duration of surgery were found to be significantly associated with intraoperative hypothermia.

## Supporting information

S1 Data (SAV)
